# Synthetic embryology of the human heart

**DOI:** 10.3389/fcell.2024.1478549

**Published:** 2025-01-28

**Authors:** Maria Belen Paredes-Espinosa, Janet L. Paluh

**Affiliations:** Department of Nanoscale Science and Engineering, College of Nanotechnology, Science and Engineering, University at Albany, Albany, NY, United States

**Keywords:** cardiogenesis, organogenesis, hiPSC, gastruloid, organoid, assembloid, neurons, ICNS

## Abstract

The evolution of stem cell-based heart models from cells and tissues to organoids and assembloids and recently synthetic embryology gastruloids, is poised to revolutionize our understanding of cardiac development, congenital to adult diseases, and patient customized therapies. Human induced pluripotent stem cell-derived cardiomyocytes (hiPSC-CMs) have already been integrated into transplantable patches and are in preclinical efforts to reverse fibrotic scarring from myocardial infarctions. To inform on the complexity of heart diseases, multi-tissue morphogenic heart models are needed that replicate fundamental components of heart function to heart organogenesis *in vitro* and which require a deep understanding of heart development. Organoid and assembloid models capture selected multicellular cardiac processes, such as chamber formation and priming events for vascularization. Gastruloid heart models offer deeper insights as synthetic embryology to mimic multi-staged developmental events of *in vivo* heart organogenesis including established heart fields, crescent formation and heart tube development along with vascular systemic foundation and even further steps. The human Elongating Multi-Lineage Organized Cardiac (EMLOC) gastruloid model captures these stages and additional events including chamber genesis, patterned vascularization, and extrinsic central and intrinsic cardiac nervous system (CNS-ICNS) integration guided by spatiotemporal and morphogenic processes with neural crest cells. Gastruloid synthetic embryology heart models offer new insights into previously hidden processes of development and provide powerful platforms for addressing heart disease that extends beyond cardiomyocytes, such as arrhythmogenic diseases, congenital defects, and systemic injury interactions, as in spinal cord injuries. The holistic view that is emerging will reveal heart development and disease in unprecedented detail to drive transformative state-of-the-art innovative applications for heart health.

## Introduction

The reproducible and exquisite choreography of staged events in embryonic development guides the molecular, anatomic and physiologic foundations of a complex organism from a fertilized egg to functional outcomes in the adult. Gastrulation enables the emerging blueprint for the morphogenesis of the embryo, establishing the major spatial axes of the adult organism via interactions of cells and tissues derived from principal germ layers of mesoderm, ectoderm and endoderm (Tam and Behringer, 1997; Arnold and Robertson, 2009). Synthetic embryology refers to *in vitro* embryo models that via stem cell gastruloid technology mimic discrete developmental stages and thus by access to multiomics provides new insights and benchmarks relevant to human health (Shao and Fu, 2020; Popovic et al., 2021). Although the full details of cell lineages, cell signaling and spatiotemporal cues in embryonic development remain nebulous, clarity at some levels regarding the intricate layers of organization and regulatory mechanisms that govern cell fate decisions and trajectories across tissues are gradually being unraveled (Shahbazi et al., 2019). Revealing these mechanisms, and their interactions, often involves manipulating genes, signals, and morphology—a task particularly challenging in mammalian embryology due to the inaccessibility of the conceptus during implantation when many critical events occur (Shahbazi and Zernicka-Goetz, 2018; Hadjantonakis et al., 2020). The field of human stem cell biology is allowing us to push the frontiers of discovery in embryology, that has long been dominated by studies in non-human mammalian model organisms. This includes determining the level of compartmentalized development that is possible *in vitro* to study organs and organ systems along with the ability of cells to recognize positional information in assembloids. At the forefront of these efforts is multisystem vascular and neural integration along with sufficient cellular and tissue diversity and patterning. Achieving these goals will enable a foundational understanding of organogenesis and organ-based diseases, advance drug discovery platforms, and bring us closer to full organ customizations.

Traditional heart embryology has relied heavily on observational methods in mammalian models, or post-mortem human studies (Mall, 1912; Davis, 1927; Streeter, 1945). Neither approach provides finite detailed insights into the dynamic spatiotemporal processes of embryogenesis or accessibility to molecular analysis or ease of perturbation studies. Typically, heart embryology has been viewed through a static set of snapshots of the embryo at different stages rather than a continuous picture of developmental events. A refined sequential and complex map of interactions that occur during cardiac development is yet to be achieved. Techniques such as single cell RNA sequencing and spatial transcriptomics are now enabling more detailed molecular mapping of gene expression patterns during cardiac development and investigation into their relevance in heart formation and disease. A comprehensive spatiotemporal atlas of the developing human heart (Asp et al., 2019) reveals the complex transcriptional landscape of cell types populating the embryonic heart at three developmental stages and maps cell-type specific gene expression to specific anatomical domains. Similarly, Cui et al. (2019) employed single-cell RNA sequencing to profile the gene expression landscapes of cardiac cells from human embryos and identified a series of unique features of human heart development by comparing gene expression profiles between humans and mice. The role of gene signatures was further expanded by generating single-cell chromatin accessibility maps of human fetal heart tissues, which identified eight major differentiation trajectories (Ameen et al., 2022). Human prenatal cardiomyocytes have also been more fully described (Sylvén et al., 2023). This study integrated single-cell RNA sequencing, spatial transcriptomics, and ligand-receptor interaction information that resulted in identification of eight types of developing cardiomyocytes. These heart cells exhibit high variability in cell cycle activity, mitochondrial content, and connexin gene expression, and are differentially distributed in the ventricles, including outflow tract, and atria, including sinoatrial node. Such findings offer deep insights into cardiac development, however the rare ability to obtain developing human heart tissue samples limits the scope of ongoing research.

Major breakthroughs in understanding the full complexity of cardiac development and associated diseases are expected through stem cell gastruloid approaches as synthetic embryology. This cradle culture biology represents developmentally accurate organogenesis *in vitro* with ability to apply unlimited resources for probing spatiotemporal patterning, morphogenesis and functional consequences, mimicking embryonic development stages (Clevers, 2016; Fu et al., 2021; Kim et al., 2023). The replication of compartmentalized partial developmental processes of embryos outside of the full natural biological context was first shown with brain cortex organoids (Qian et al., 2019), but perhaps never so fully in regard to more complete organogenesis as with gastruloid cardiogenesis models (Rossi et al., 2021; Olmsted and Paluh, 2022). Together these gastruloid heart models reveal the emergence of heart fields, heart tube formation, vascular foundations and more extensive chamber genesis with contractility, conduction and neuronal systemic connections. Synthetic embryology applied to cardiogenesis and organogenesis provides a platform embracing development for studying congenital to adult heart diseases. The new heart gastruloid capabilities, along with a wealth of partially structured organoid and assembloid models that have led the way, provide enormous capabilities to monitor and perturb cardiogenic events in real time to recreate and study disease states and elaborate underlying mechanisms and solutions in a controlled environment. This includes congenital structural defects, myocardial infarctions, arrhythmias, and other functional or injury related abnormalities. Here we discuss human synthetic embryology for cardiogenesis through the lens of human heart development, human stem cell biology and its foundational heart models including cells, tissues, organoids and assembloids, alongside advancements in bioengineering. Human stem cell heart technologies are already making their way into clinical trials for translational impacts on human cardiac health, with the next revolution of transformative advances via synthetic embryology models making their debut.

## Development of the human heart

### Morphogenic transition of heart fields to loop events

**FIGURE 1:**
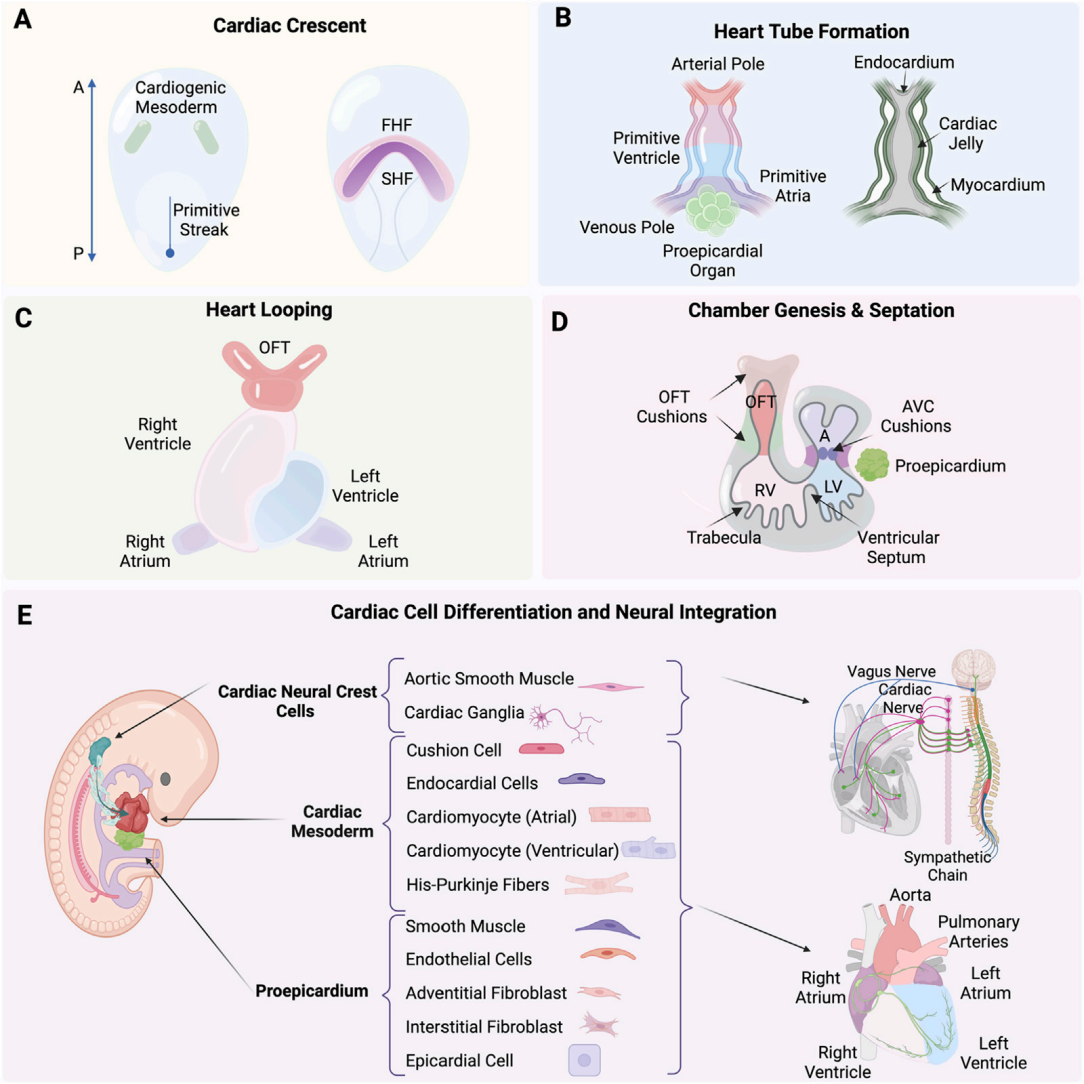
**(A)** Formation of the cardiac crescent from the cardiogenic mesoderm, including the first heart field (FHF) and second heart field (SHF). **(B)** Formation of the heart tube from the arterial pole to the venous pole, including the primitive ventricle, atria, and the proepicardial organ, with the endocardium, cardiac jelly, and myocardium layers. **(C)** Process of heart looping. **(D)** Septation and the formation of heart chambers, including the outflow tract (OFT) cushions, atrioventricular (AVC) cushions, right ventricle (RV), left ventricle (LV), and trabecula. **(E)** Differentiation of cardiac neural crest cells (cNCCs), proepicardium, and cardiac mesoderm into various cardiac cells and their neural integration. Created with BioRender.

The formation of the human heart is driven by precisely timed and regulated events initiated at gastrulation at the end of the second week of human development (Carnegie Stage 7; O’Rahilly and Muller, 1987). As such, it is one of the first organs to form and function during embryogenesis. Concurrently, vasculogenesis establishes the earliest vascular endothelial cells, as mesodermal cells differentiate directly into endothelial precursors, forming a primitive vascular plexus that supports the emerging heart and other embryonic structures (Patan, 2000). Extensive studies have primarily used avian and mouse models because of the easily accessible embryo, contributing significantly to our understanding of cardiogenesis, summarized here and explained in great detail in (Buijtendijk et al., 2020). Mesp1 is expressed the earliest along the primitive streak and marks the cardiac mesodermal population that gives rise to both the primary and the secondary heart fields (Saga et al., 1996). The determination process begins with the formation of the primary heart field from mesodermal cells at the embryo’s anterior, which differentiate into cardiac progenitor cells (Figure 1A) (Garcia-Martinez and Schoenwolf, 1993; Tam et al., 1997). The straight heart tube is a consequence of the ventral midline fusion of myocardial and endothelial cells. These emerge from the bilateral primary heart fields in the lateral plate mesoderm, in a process that is initiated by an inductive signal from the anterior endoderm (Schultheiss et al., 1995). The heart tube consists of an outer myocardium and inner endocardium separated already by a layer of cardiac jelly (Figure 1B) (Davis, 1924). At the site of gastrulation, Wnt growth factors block differentiation of the mesodermal cells. As these cells migrate anteriorly, they leave the Wnt expression domain and enter a domain of active Wnt inhibition, gaining the capacity to enter the cardiac lineage. Next steps are coordinated by Bone morphogenetic protein (BMP) and growth factors secreted by endodermal and ectodermal cells at the lateral border of the flat embryo. Cardiomyocytes appear at the beginning of the third week of development (Carnegie Stage 8; O’Rahilly and Muller, 1987) when precardiac mesodermal cells differentiate into this specialized contractile muscle cell (Marvin et al., 2001; Schneider and Mercola, 2001). BMP inhibitors secreted by the neural tube regulate the medial expansion, whereas FGF growth factors expressed by the endoderm determine the posterior border of the heart-forming region (Waldo et al., 2001). Beginning in the fourth week of development (Carnegie Stage 10; O’Rahilly and Muller, 1987), the initially straight and bilaterally symmetric heart tube transforms into a convoluted heart loop. The morphogenic process of looping defines the future cell positional relationships and leads to the formation of the primitive atrium, ventricle and outflow tract (OFT) (for review see Männer, 2009). It is the rupture of the dorsal mesocardium along its midline that causes the heart tube to undergo a single counterclockwise winding and torsion around its center axis to form a “C-shaped” loop (Männer, 2004). The elongating tube bends and coils further into a more complex helical “S-shaped” loop, involving a left-handed helix caudally and a right-handed helix cranially, termed a two-handed helix or helical perversion (Figure 1C) (Männer, 2013). Differential cell growth, cardiac jelly swelling and dorsal mesocardium tension, together, play active roles in directing changes, with the myocardial shape playing a secondary role (Shi et al., 2014).

The heart tube increases at both arterial and venous poles five-fold in length by the continuous addition of newly differentiated cardiomyocytes. A rapidly proliferating pool of precursor cells from the pharyngeal mesoderm, known as the second heart field (SHF) contribute (Soufan et al., 2006), mediated through the canonical Wnt/β-catenin signaling (Norden and Kispert, 2012; Ruiz-Villalba et al., 2016). These SHF progenitor cells express the transcription factor Islet1 as they are incorporated into the heart tube. As the cells differentiate into cardiomyocytes, they cease proliferation, marked by the downregulation of Islet1 and the upregulation of Nkx2.5 (Cai et al., 2003). The balance between proliferation and differentiation is tightly controlled in space and time by a network of signaling pathways and downstream transcription factors, in which the T-box factor Tbx1 plays a particularly prominent role in directing SHF cells to the inflow and outflow poles of the heart (Rana et al., 2014; De Bono et al., 2018).

### Heart tube to chamber genesis

A single cavity exists after heart tube looping, not yet divided into chambers. What follows is a series of spatial and temporal cell diversification, morphogenic and signaling events that will define chambers and lay the framework for systemic connections. This includes the intrinsic nervous system as well as angiocrine roles enabled in part by heart endothelial cells (Kim et al., 2021). Septation defines the lower ventricular chambers, closing off a temporary opening that is the primary interventricular foramen. The left and right ventricles develop from the inlet or outlet part of the ventricular loop, respectively, using a process referred to as ballooning. These outpouchings at the outer curvature are crucial for the ventricles’ morphological development and is marked by the appearance of myocardial trabeculations, which provide the ventricles their distinct identities (Christoffels et al., 2000). Cardiac jelly is initially present between the endocardial and myocardial layers but disappears. Trabeculations appear on the endocardial inner chamber surface (Figure 1D). The ventricular cardiomyocytes are also developing and begin to express key genes such as atrial natriuretic factor (Anf/Nppa) and the gap-junction protein Connexin 40 (Gja5) (Houweling et al., 2002) along with the T-box transcription factors Tbx5 and Tbx20 that play crucial activating roles in developing ventricular cardiomyocytes. In contrast, Tbx2 and Tbx3 act as important repressors, inhibiting the ventricular myocardium gene programto define the atrioventricular region (Greulich et al., 2011; Habets et al., 2002). During events of ventricular development and heart tube lengthening, the atria begin to differentiate at the inflow region (Yutzey et al., 1994; Christoffels et al., 2000). The superolateral walls of the primary atrial component balloon out on either side of the outflow tract and form the left and right atrial appendages. This marks the first step to distinguish morphologically and functionally the two sides of the primary atrium (Christoffels et al., 2000; Harvey, 2002), including a crucial role for Pitx2 plays in establishing heart left-right asymmetry (Mommersteeg et al., 2007).

### Chamber septation and tissue diversification for vascular integration

Chamber septation and expansion of the cardiac jelly in both the atrioventricular canal (AVC) and the outflow tract (OFT) are coincident. The cardiac jelly is shaped into four major cushions that are the posterior and anterior atrioventricular cushions in the AVC, and the parietal and septal outflow cushions, often referred to as ridges, in the OFT (Figure 1D) (Markwald et al., 1977). Initially, these cushions are populated by mesenchymal cells derived from the endocardium through an endocardial-to-mesenchymal transition (EndMT), a process initiated by Notch and promoted by BMP and TGFβ signaling pathways (reviewed in Garside et al., 2013). In addition to EndMT-derived cells, the OFT also becomes populated by cardiac neural crest cells (cNCCs), which migrate into these cushions and form the condensed mesenchyme at the junction where the future subaortic and subpulmonary myocardium will develop (Figure 1E) (Kirby et al., 1983; Waldo et al., 1998). The condensed mesenchyme known as the aorticopulmonary septation complex divides the distal OFT into the bases of the aorta and pulmonary trunks and the middle outflow tract into the aortic and pulmonary semilunar valve regions. This leads to the eventual division of the OFT into the pulmonary artery and aorta by the conotruncal septum (Waldo et al., 1998; 1999). The cushions expand and grow towards each other, eventually meeting and fusing, thereby separating the left and right bloodstreams and contributing to the formation of the atrial and ventricular septa. Proper alignment and fusion of these structures are crucial for developing a functional four-chambered heart, capable of efficiently directing oxygenated and deoxygenated blood through the systemic and pulmonary circulations (reviewed in Moorman et al., 2003; Moorman and Christoffels, 2003).

### Epicardium and coronary vasculature formation

The epicardium, derived from the proepicardial organ, is a crucial contributor to heart development. During early heart development, the proepicardial organ appears as a cluster of cells adjacent to the developing heart tube. Epicardial cells migrate over the surface of the heart tube to form a continuous epithelial sheet around it. Subsequently, these cells undergo an epithelial-to-mesenchymal transition (EMT) to populate the subepicardial space, where they give rise to epicardial-derived cells (EPDCs) (Figure 1E). The transcription factors T-box18 (Tbx18) and Wilms’ tumor 1 homolog (Wt1), bi-directionally control the epicardial EMT through their effects on Slug expression (Takeichi et al., 2013). EPDCs differentiate into various cell types, including fibroblasts, smooth muscle cells, and endothelial cells, which are essential for the formation of the coronary vasculature and interstitial fibroblasts (Dettman et al., 1998; Pérez-Pomares et al., 2002). This process is tightly regulated by signaling pathways involving key molecules such as TGF-β, BMP, FGF, and Wnt, which orchestrate the proliferation, migration, and differentiation of EPDCs. Epicardial signaling is essential for myocardial growth, as it provides critical paracrine signals that stimulate the proliferation and maturation of cardiomyocytes. The formation of the coronary vasculature begins around the fourth week of human development (Carnegie Stage 10; O’Rahilly and Muller, 1987), facilitated by EPDCs differentiating into endothelial and smooth muscle cells, thereby forming the blood vessels that will supply the growing myocardium with oxygen and nutrients (Gittenberger-De Groot et al., 1998; Lavine et al., 2006; Perez-Pomares and De La Pompa, 2011). Additionally, the interaction between epicardial cells and myocardial cells is crucial for the proper patterning and maturation of the coronary vessels, ensuring that the myocardium receives an adequate blood supply to support its increasing metabolic demands during development (Lavine et al., 2006).

### Cardiac neural crest cells and tissue diversification for neural integration

Some of the earliest descriptions of cardiac neural crest cells (NCCs), map their origin from the dorsal neural tube where they delaminate and migrate along predetermined pathways to the heart, reaching pharyngeal arches 3, 4, and 6 (Figure 1E) (Kirby et al., 1983; Phillips et al., 1987). Multiple studies are defining the integral roles of cardiac NCCs in heart development and systemic function, including contributing to the origin of numerous cell types for roles in heart septation, aortic arch arteries, aorticopulmonary septum, cardiac valves (Kirby et al., 1983), connective tissue insulation of the His-purkinje conduction system (Gurjarpadhye et al., 2007), and parasympathetic innervation to the heart (Kirby and Stewart, 1983). The migration and differentiation of cardiac NCCs define the cellular spatial context during morphogenesis through a network of signaling pathways including BMPs, FGFs, NOTCH, and WNT. These factors guide the establishment of the neural plate border, and the cytoskeletal rearrangements required for the delamination and migration of NCCs from the neural tube, ensuring proper cardiac development (Scholl and Kirby, 2009). The molecular subcircuit that confers cardiac NCCs with their unique ability to contribute to the heart is comprised of transcription factors Sox8, Tgif1, and Ets1 (Gandhi et al., 2020). Pax3 has also been found to play a crucial role in the early development of cardiac NCCs. Its deletion can lead to defects in the formation of cardiac structures including the improper development of the cardiac outflow tract and aortic arch arteries, resulting in congenital heart defects (Franz, 1989). Similarly, mutations or deletions in Edn1 disrupt migration and differentiation of cNCCs leading to severe malformations such as interrupted aortic arch and defects in the septation of the heart (Kurihara et al., 1999).

### Integration of the intrinsic cardiac nervous system (ICNS) with the central nervous system (CNS)

The functional connections necessary to regulate heart rate, rhythm, and force of contraction rely on. Co-development of the heart’s intrinsic nervous system (ICNS), with myocardial and vascular tissues and the central nervous system. The ICNS network of neurons contains afferent (sensory), interconnecting (local circuit), and cardio-motor (efferent sympathetic and parasympathetic) neurons. ICNS neurons interact with the extracardiac thoracic ganglia, both of which are regulated by the CNS (Figure 1E). Nearly every aspect of cardiac electrophysiology, conduction and contraction described as chronotropy (heart rate), inotropy (contractility), lusitropy (rate of relaxation), and dromotropy (conduction) are fine-tuned by the autonomic nervous system (ANS). Neural processing and nested feedback loops occur at multiple levels and within the pericardium and extracardiac ganglia of the heart and also establish broader CNS connections to the spinal cord and brain (Fukuda et al., 2015; Fedele and Brand, 2020; Ripplinger, 2022). The migration and differentiation of NCCs forms the cardiac ganglia as well sympathetic and parasympathetic ganglia. The NCCs that will form parasympathetic neurons delaminate from the neural folds at the level of the hindbrain. They then migrate along tracts laid down by the vagus nerve, entering the heart and forming ganglia closely associated with the epicardial fat. Parasympathetic innervation is established and functional before sympathetic innervation. Glial cell line-derived neurotrophic factor (GDNF) and neurturin are essential for the survival and patterning of parasympathetic neurons. On the other hand, sympathetic neurons originate from NCCs that migrate to form the paravertebral sympathetic stellate ganglia. Sympathetic axons extend to the heart and target various cardiac tissues including the pacemaker cells, atrial and ventricular myocardium. The development of sympathetic neurons is heavily dependent on neurotrophic factors such as nerve growth factor (NGF) and neurotrophin-3 (NT-3). In addition, artemin, a member of the GDNF family, and endothelin-3 are also important in sympathetic axonal guidance to their targets. The correct migration and differentiation of NCCs ensure the formation of both sympathetic and parasympathetic ganglia, which are crucial for establishing effective and precise innervation patterns in the heart (Hildreth et al., 2009; Hasan, 2013).

## Stem cell models of cardiogenesis: from cells to synthetic embryology

### Foundational hiPSC-CMs in drug studies and tissue repair for clinical benefit

Compelling advances for the use of stem cell derived heart cells and tissues in human health begins with hiPSC-CMs that have seen considerable advancement from *in vitro* assays to clinical trials. The extreme ease to generate hiPSC-CMs (for review see Lyra-Leite et al., 2022) and their wide commercial availability provides a valuable rapid and unlimited resource for developing CM-focused assays and CM-directed therapies. Translational applications include regenerative studies (Shiba et al., 2014; Ye et al., 2014; Gao et al., 2018; Zhao et al., 2021) and detailed drug screening studies (Tanaka et al., 2009; Liang et al., 2013; Shinozawa et al., 2017; Blinova et al., 2018; Zeng et al., 2019). One of the earliest applications of hiPSC-CMs was in drug screening, where (Tanaka et al., 2009) highlighted their ability to detect electrophysiological changes, including action potential alterations caused by inhibitors of sodium, calcium, and potassium ion channels. Patient-specific hiPSC-CMs were used in a scaled drug study to predict cardiotoxicity and identify variance in drug susceptibilities across multiple heart disease backgrounds (Liang et al., 2013). The study applied a library of hiPSC-CMs from healthy individuals and patients with hereditary cardiac disorders, including long QT syndrome, hypertrophic cardiomyopathy, and dilated cardiomyopathy. Electrophysiology changes, including action potential duration and arrhythmias were quantified. Individual patient susceptibility to drugs was further studied by (Shinozawa et al., 2017) applying hiPSC-CMs to model individual susceptibility to moxifloxacin-induced QT prolongation. Among ten healthy patients a strong correlation was revealed between *in vitro* field potential duration and *in vivo* QT prolongation, demonstrating utility of patient hiPSC-CMs for preclinical cardiotoxicity testing. In a standardized study across ten sites (Blinova et al., 2018), validated hiPSC-CMs for predicting torsades de pointes (TdP) risk, using both multi-electrode array (MEA) and voltage-sensing optical (VSO) technologies. Twenty-eight drugs with varying TdP risk profiles were evaluated. The study findings highlighted the reproducible potential of hiPSC-CMs’ for improving drug safety and specificity in preclinical cardiotoxicity testing. Pharmacological responses in hiPSC-CMs revealed sex- and ethnicity-related differences, as demonstrated by (Zeng et al., 2019). Using cells from diverse donors (Caucasian, Black, Asian Indian, Asian), they tested eight drug classes, to evaluate electrophysiological changes such as action potential alterations and early afterdepolarizations. The hiPSC-CMs from female donors revealed higher sensitivity to hERG channel blockers that varied across ethnic backgrounds. The number of *In vitro* hiPSC-CMs studies have expanded significantly and include numerous additional cardiac conditions that include hypertrophic cardiomyopathy (Cashman et al., 2016; Lam and Wu, 2018), arrhythmogenic right ventricular cardiomyopathy (Caspi et al., 2013; Kim et al., 2013; El-Battrawy et al., 2018), long QT syndrome (Moretti et al., 2010; Itzhaki et al., 2011; Jouni et al., 2015; Rocchetti et al., 2017; Wuriyanghai et al., 2018; Takaki et al., 2019), and dilated cardiomyopathy (Sun et al., 2012; Wyles et al., 2016; Dai et al., 2020). Several focused reviews on hiPSC- CMs further detail the scope of hiPSC-CM applications and bioengineering advances (Di Baldassarre et al., 2018; Mummery, 2018; Nakao et al., 2020).

Evaluation of the regenerative capacity of hiPSC-CMs in animal models to repair myocardial infarction (MI) damage by replacing fibroblastic tissue with functional cardiomyocytes represents a significant preclinical effort. MI leads to significant cardiomyocyte loss due to ischemic injury, primarily resulting from coronary artery atherosclerosis (Reed et al., 2017). This injury typically results in the replacement of functional cardiomyocyte tissue with non-contractile fibrotic scar tissue, which can encompass a substantial portion of the infarcted region (Talman and Ruskoaho, 2016). Given that adult cardiomyocytes exhibit minimal regenerative capacity, research has focused on cell therapy as a viable strategy to restore cardiac function. Several promising preclinical *in vivo* regenerative animal studies with hiPSC-CMs in bioengineered patches indicate that repair of heart tissue damaged by myocardial infaction is possible through a variety of platforms. Research by Ye et al. (2014) demonstrated that hiPSC-CMs on fibrin-based patches were effective in enhancing left ventricular function and reducing fibrosis in a porcine model. In a primate model with allogeneic hiPSC-CMs, Shiba et al. (2016) demonstrated that cells survive without immune rejection, electrically integrate with the host myocardium, and improve heart function, although a risk of transient ventricular tachycardia was noted. Further advancements were made by Gao et al. (2018), who engineered large cardiac muscle patches from hiPSC-CMs, smooth muscle cells, and endothelial cells within a fibrin scaffold. These patches improved myocardial recovery in a swine infarction model, reduced wall stress, and achieved synchronized contractions. In another porcine myocardial infarction study Zhao et al. (2021) demonstrated that overexpression of the G1/S cyclin D2 in hiPSC-CMs enhanced their engraftment, proliferation, and the vascular density within the transplanted cardiomyocytes, which led to better left ventricular function and reduced fibrosis in a porcine myocardial infarction model.

Human studies on the safety and efficacy of hiPSC-CM therapies are also being evaluated in a growing number of clinical trials for severe heart failure, employing various delivery methods to optimize their safety and efficacy. The BioVAT-HF trial (NCT04396899, 2020) in Germany tests engineered human myocardium (EHM) of hiPSC-CMs (800 million) and stromal cells embedded in a bovine collagen hydrogel as a ventricular assist tissue on patients with severe heart failure in the left ventricular ejection fraction (LVEF) < 35%). Primary outcomes focus on echocardiography and cardiac MRI over 12 months. Initial findings support safety, vascularized remuscularization, heart wall thickening and improved heart function (Ensminger et al., 2024). Other human trials are ongoing. The HEAL-CHF trial (NCT03763136, 2021) in China targets severe chronic ischemic cardiomyopathy (LVEF 20%–45%) by epicardial injection of hiPSC-CMs (200 million) delivered during coronary artery bypass grafting (CABG). Primary outcomes focus on ventricular arrhythmias and tumor incidence up to 6 months post-operation. Another China trial (NCT04982081, 2021) explores catheter-based endocardial injection, delivering hiPSC-CMs (HiCM188; 100 to 400 million) to patients with severe congestive heart failure (LVEF <40%). Primary outcomes focus on adverse events, with secondary outcomes assessing ventricular function, functional status, and tumor formation. The LAPiS Study (NCT04945018, 2022) in Japan evaluates hiPSC-CM spheroids (HS-001) in patients with severe ischemic heart disease (LVEF <40%) in a Phase I/II dose-escalation study. Primary outcomes focus on safety and tolerability over 26 weeks, and secondary outcomes including cardiac function, myocardial blood flow, physical performance, and quality of life. Collectively, these trials contribute critical data on the feasibility, safety, and efficacy of hiPSC-CM-based therapies in advanced heart failure.

New frontier applications for hiPSC-CM research includes off-planet low gravity experiments relevant to Space travel (Wnorowski et al., 2019; Rampoldi et al., 2022; Hwang et al., 2023). In contrast to embryos that require gravity during embryogenesis to establish polarized structures (Duprat et al., 1998; Ruden et al., 2018), hiPSC-CMs can be used to inform on molecular and physiologic adaptation. This includes long-term spaceflight and irradiation with the goal to identify potential countermeasures relevant for astronauts and life in Space. Changes in cardiomyocyte structure and function under microgravity conditions include alterations in gene expression (Hwang et al., 2023), calcium handling (Liu et al., 2020), and contractility (Liu et al., 2020; Acharya et al., 2022; Hwang et al., 2023). Observed effects of long-term space microgravity on gene expression in hiPSC-CMs includes genes and pathways relevant to cell proliferation, differentiation, and cardiac function, including upregulation of genes such as CCBE1, CCND2, IGFBP5, and BDKRB2. Pathways such as TGF-beta and adrenergic signaling, along with protein kinase A signaling, were also upregulated, contributing to improved calcium cycling, muscle contractility, and overall cardiac function. Additionally, genes including MYL2, TNNI3, RYR2, TCAP, SCN5A, ATP2B4, PRKACA, and ANK2 were notably upregulated, enhancing muscle contraction and cardiac conduction. Conversely, genes associated with extracellular matrix (ECM) regulation and focal adhesion, such as ITIH5, COL4A4, and PTPR21, ITGA11, and ANXA1, were downregulated, indicating reduced ECM and adhesion activities. This modulation suggests potential benefits of microgravity for CM applications in cardiac regeneration and spaceflight-related health countermeasures (Wnorowski et al., 2019; Hwang et al., 2023). The foundational studies with hiPSC-CMs highlight the significant and extensive discoveries possible with even 1 cell type of the heart. Expanded opportunities beyond hiPSC-CM models for cardiac research, particularly more complex heart models via synthetic embryology are expected as these technologies and platforms to study them evolve.

### Foundational partially structured heart models capture specific cardiac processes

**FIGURE 2:**
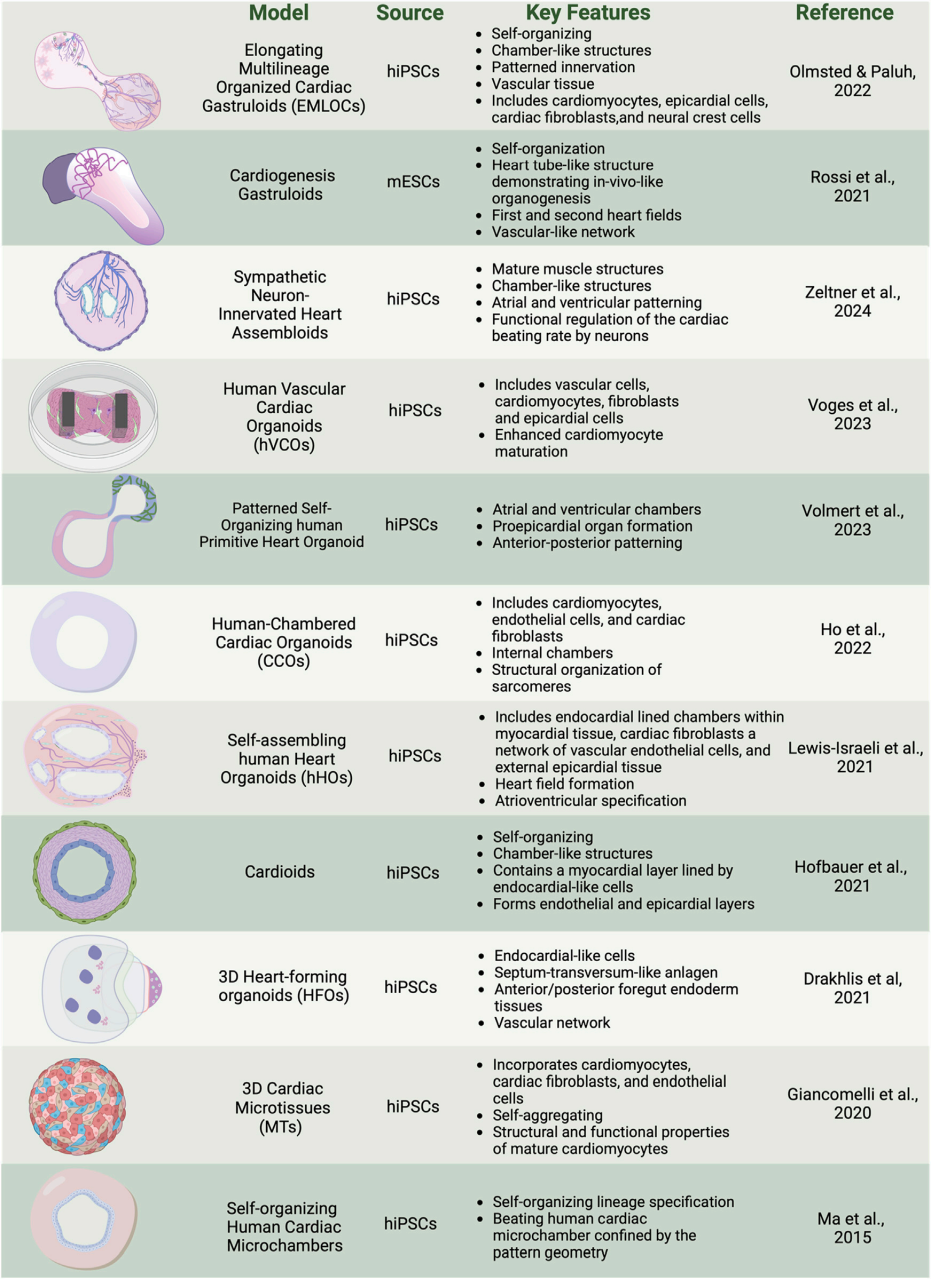
Overview of cardiac organoid and gastruloid models. *In vitro* 3D heterogenous cell culture systems include most advanced gastruloids as models of synthetic embryology, and partial organogenesis models depicted in organoids, and assembloids. Created with BioRender.

Developmental biology builds the foundational components necessary to recapitulate key decision points to drive self-assembly for organogenesis in human multicellular heart models. Human stem cell organoids and assembloids focus on partial organogenesis but do not implement full self-organizing requirements or tissue complexity that is achieved via activation of all three germ lineages as in more natural gastruloid protocols, the latter so named due to their biomimicry of embryogenesis. Several recent reviews discuss the general evolution of organoid types of stem cell models (Kim et al., 2022; Roshanravan et al., 2023). We briefly summarize here some of the unique applications of organoids that emphasize patterned bioengineering and self-aggregating freely forming microtissues, human heart forming organoids in matrigel, self-organizing cardioids, and other self-assembling human heart organoids (Figure 2). These models capture components of the complexity of synthetic embryology to provide a range of insights into heart function.

There is significant interest in how bioengineered platforms can be used to direct self-organization of cardiac structures, such as microchambers for drug toxicity screening. In a geometric confinement study, hiPSCs were plated on poly (ethylene glycol) (PEG)-patterned substrates in a tissue culture dish. A polydimethylsiloxane (PDMS) stencil allowed precise etching of the desired pattern onto tissue culture polystyrene, applying oxygen plasma, and plating a film of PEG. The PEG-retained hiPSCs are restricted to patterned outgrowth. hiPSC cardiac differentiation was done by modulation of the WNT/β-catenin pathway with the GSK3 inhibitor CHIR99021 to induce mesoderm differentiation and spatial organization on the PEG. Increased proliferation at the perimeter was associated with mechanical stress and RhoA pathway activation. Larger patterns supported the formation of beating cardiomyocytes in the center, surrounded by myofibroblasts, creating three-dimensional (3D) cardiac microchambers. The two-cell type layered microchamber organization is reminiscent of early cardiac development, and platforms were used to test drug toxicity screening with thalidomide, showing reduced differentiation efficiency and impaired microchamber function (Ma et al., 2015; Hoang et al., 2018).

Self-aggregating cardiac microtissues (MTs) incorporating multiple cell types of cardiomyocytes, cardiac fibroblasts, and endothelial cells from hiPSCs have been generated separately and combined in scaffold-free systems to self-organize. Microtissues constructed from hiPSC-derived cardiomyocytes (hiPSC-CMs), cardiac endothelial cells (hiPSC-ECs), and cardiac fibroblasts (hiPSC-CFs) were differentiated separately using specific growth factors and signaling inhibitors: Activin A, BMP4, and CHIR99021 for cardiac mesoderm induction, VEGF and XAV 939 for endothelial cells, and FGF-2 for fibroblasts. Cells were combined in a fixed ratio (70% CMs, 15% ECs, 15% CFs) and self-aggregated into microtissues in V-shaped 96-well plates. The CMs within the microtissues begin spontaneous beating within a few days and mature over 21 days, displaying structural and functional properties of mature CMs including sarcomere organization, contraction duration and amplitude, action potential profiles, and expression of postnatal sarcomere isoforms. Functional assays, including video analysis of contraction and calcium transients, Seahorse metabolic assays for mitochondrial and glycolytic activity, and sharp electrode recordings for electrophysiological properties, demonstrate the enhanced maturation of hiPSC-CMs within these microtissues. The protocol also supports the cryopreservation of differentiated cells, enabling consistent microtissue formation from banked stocks (Giacomelli et al., 2020; Campostrini et al., 2021).

**FIGURE 3:**
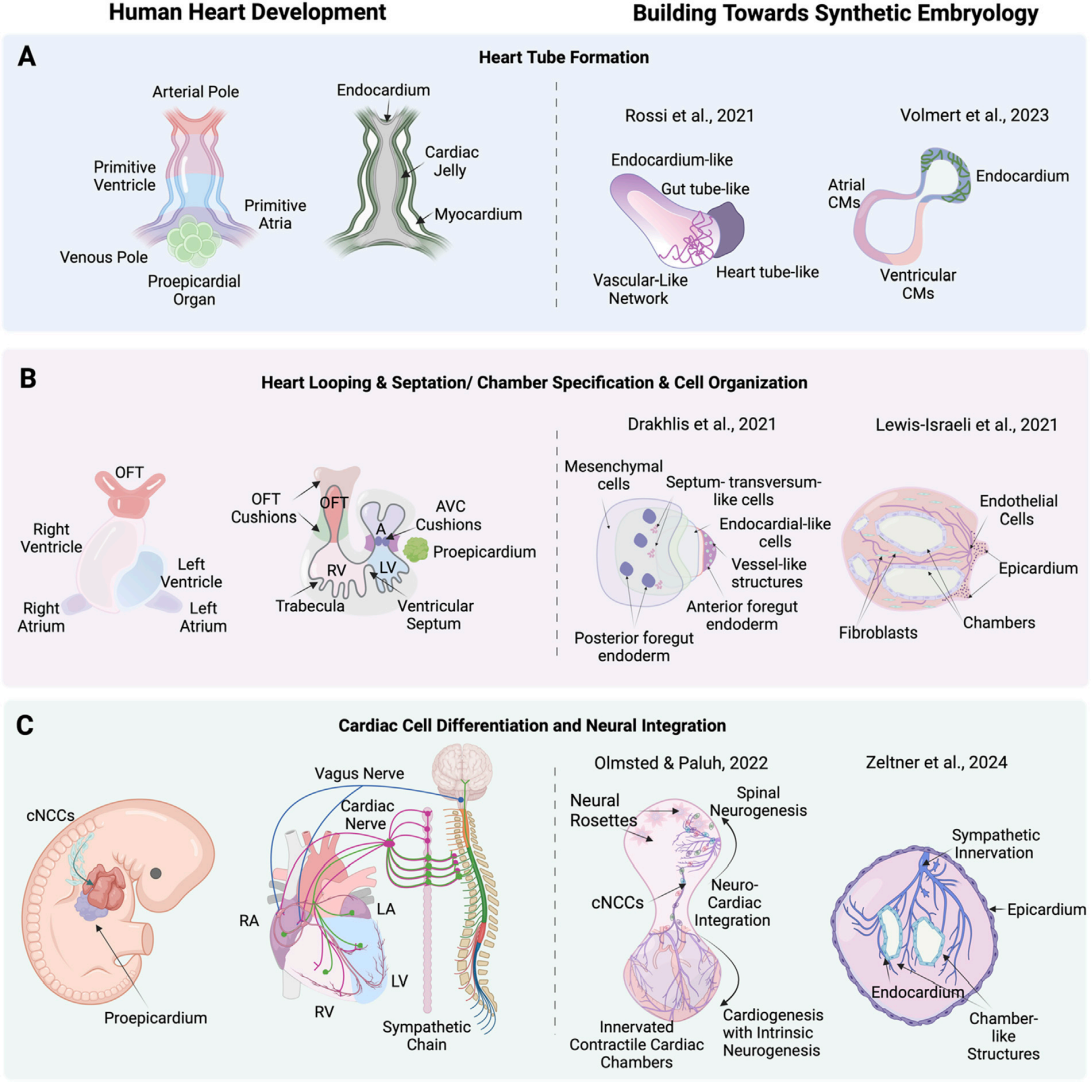
Stages of human heart development alongside corresponding advanced cardiac stem cell models including synthetic embryology approaches. **(A)** heart tube formation in human heart development, and corresponding, cardiac stem cell models by Rossi et al. (2021) (mouse) a synthetic embryology approach and Volmert et al. (2023) depicting endocardium-like structures, gut tube-like formations, and vascular networks that mimic heart tube-like structures. **(B)** Heart looping, septation, and chamber formation in human heart development, and stem cell models by Drakhlis et al. (2021) and Lewis-Israeli et al. (2021) showing mesenchymal cells, septum-transversum-like cells, endocardial-like cells, and chamber structures. **(C)** Cardiac cell differentiation and neural integration in human heart development, showing the differentiation of cardiac neural crest cells (cNCCs) and their integration with cardiac mesoderm and proepicardium, forming various cardiac cells and nerves and corresponding neuronal integration models by Olmsted and Paluh (2022) exemplifying synthetic embryology through neuro-cardiac integration and sympathetic innervation and Zeltner et al. (2024) highlighting neural rosettes, and sympathetic innervation in cardiac chambers. Created with BioRender.

Human heart-forming organoids (HFOs) in Matrigel have also been described and include components of early heart and foregut development. hiPSC aggregates in Matrigel are directed to cardiac differentiation using biphasic WNT pathway modulation with CHIR99021 and IWP2. The structure of HFOs resembles early heart development stages before the heart tube forms, which is known to require interaction with the foregut endoderm. The resulting organoids exhibit a complex structure with a myocardial layer lined by endocardial-like cells, surrounded by septum-transversum-like cells, and containing distinct anterior and posterior foregut endoderm tissues (Figure 3B). Cardiomyogenesis was monitored using NKX2.5-eGFP, SOX17, SOX2, and HNF4α to identify anterior and posterior foregut endoderm. CD31 staining was used to reveal endothelial cells forming vascular-like networks, and WT1 confirmed the presence of septum-transversum-like cells. HFOs were used to study genetic defects, with NKX2.5-knockout organoids displaying phenotypes similar to cardiac malformations observed in transgenic mice, such as decreased cardiomyocyte adhesion and hypertrophy (Drakhlis et al., 2021).

Chamber genesis has also been studied with self-organizing cardioids from hiPSCs, focusing on specification into cardiac mesoderm and CMs and intrinsic patterning and morphogenesis into chamber-like cavity structures. Key cardiogenic signaling pathways, including ACTIVIN, bone morphogenetic protein (BMP), fibroblast growth factor (FGF), retinoic acid (RA), and WNT are applied, directing the specification of hiPSCs into mesoderm, cardiac mesoderm, and beating cardiomyocyte progenitors in 2D culture. Supplementing the media with selected extracellular matrix (ECM) proteins such as laminins 521/511 facilitated the self-assembly of cells into hollow, beating 3D structures expressing CM markers TNNT2, ACTN2, TNNI1, MYL7, TTN, NPPA, and ATP2A2. The cardioids exhibit a myocardial layer lined by endocardial-like cells and can form endothelial and epicardial layers, as confirmed by the expression of biomarkers such as WT1, ACTA2, CDH5, and PECAM1. The study explores the role of the WNT-BMP signaling axis in cavity morphogenesis by demonstrating that varying the dosage of WNT during mesoderm induction can control cavity formation. High doses of WNT signaling promote cavity expansion and cardiomyocyte specification. HAND1, a transcription factor, is identified as a critical downstream target of BMP signaling necessary for proper chamber formation. HAND1 knockout cardioids exhibit reduced cavity size and impaired self-organization, indicating its essential role in the morphogenesis of cardiac structures. Additionally, cryoinjury experiments demonstrate that cardioids can model early regenerative and fibrotic responses, showing localized extracellular matrix accumulation and recruitment of fibroblast-like cells (Hofbauer et al., 2021).

Chamber genesis with vascular integration has been explored in self-assembling human heart organoids (hHOs) generated through a three-step Wnt signaling modulation strategy (Figure 3B). Internal chambers with well-organized multi-lineage cardiac cell types and complex vasculature are formed from hiPSCs by manipulating cardiac developmental programs with sequential Wnt signaling modulation (activation/inhibition/activation). The process begins with hiPSCs embryoid bodies, followed by mesoderm and cardiogenic mesoderm induction using CHIR99021 and Wnt-C59. The presence of multiple cardiac cell types, including CMs (TNNT2+), endothelial cells (PECAM1+), cardiac fibroblasts (THY1+), and epicardial cells (WT1+, TJP1+), were confirmed through immunofluorescence and transcriptomic analysis. The presence of both heart fields was evidenced by immunofluorescence that showed well differentiated regions of HAND1 and HAND2, in the same organoid. Similarly, hHOs include atrioventricular specification with Ventricular CMs (MYL2+) and Atrial CMs (MYL7+). Structural and functional features akin to fetal human hearts included sarcomere formation, action potential profiles, and calcium transients. A robust interconnected network of endothelial cells (PECAM1+), and vessel-like tube formation is seen throughout hHOs. By culturing the hHOs in diabetic-like condition of high glucose and insulin, the model was used to investigate pregestational diabetes (PGD) and revealed structural defects and altered metabolic profiles that mimic congenital heart disease phenotypes (Lewis-Israeli et al., 2021).

Chambered cardiac hiPSC organoids (CCOs) developed for cardiovascular disease modeling have been generated via self-assembly by developmentally relevant sequential differentiation. Wnt/β-catenin signal manipulation is used along with key growth factors for cardiac progenitor expansion, including Activin A and BMP4, to induce mesodermal differentiation and the sequential formation of cardiac progenitors into cardiomyocytes, endothelial cells, and cardiac fibroblasts. The presence and functionality of these cell types were confirmed through immunofluorescence staining and single-cell RNA sequencing. The CCOs developed internal chambers and exhibited CM beating, structural organization of sarcomeres, and electrophysiological properties akin to the human heart. Notably, the study highlighted the maturation of CMs over time, as evidenced by the increased expression of maturation markers, ion channel genes, and a metabolic shift from glycolysis to fatty acid β-oxidation. The CCOs successfully modeled cardiac hypertrophy by exhibiting thickened chamber walls, reduced fractional shortening, and increased myofibrillar disarray upon treatment with endothelin-1 (EDN1). Electrophysiological assessments revealed a tachyarrhythmic phenotype characterized by rapid depolarization before complete repolarization, mimicking clinical observations of hypertrophic cardiomyopathy (Ho et al., 2022).

Among the most advanced self-assembling human heart organoids are those generated through a three-step Wnt signaling modulation strategy that includes metabolic and hormonal factors. Internal chambers with well-organized multi-lineage cardiac cell types develop that closely resemble human fetal cardiac tissues (Figure 3A). The protocol involves mimicking *in utero* gestation by adding metabolic and hormonal factors, such as fatty acids (oleic acid, linoleic acid, and palmitic acid), L-carnitine, T3 hormone, and IGF-1, to induce physiological and anatomical relevance. Transcriptional and morphological similarity has been validated to age-matched human embryonic hearts, including large atrial and ventricular chambers, proepicardial organ formation, and retinoic acid-mediated anterior-posterior patterning, closely mimicking the developmental processes of the post-heart tube stage primitive heart. Key growth factors used include Activin A, BMP4, and retinoic acid. The presence of CMs, endothelial cells, and epicardial cells, was confirmed through immunofluorescence staining and single-cell RNA sequencing. The formation of distinct atrial and ventricular chambers was verified by specific markers: NR2F2, TBX5, NPPA for atrial chambers, and MYL3, HEY2, IRX4, HAND1 for ventricular chambers. The study evaluated the effects of ondansetron, a drug associated with congenital heart defects. Ondansetron-treated organoids displayed structural and electrophysiological defects, mimicking congenital heart conditions observed in humans (Volmert et al., 2023).

Human vascular cardiac organoids (hVCOs) are assembloids generated by combining 20% vascular cells with 80% hiPSC-derived cardiac cells, in a ratio of 0.2:0.6:0.2 for vascular cells, cardiomyocytes, and fibroblasts plus epicardial cells. The differentiation protocols involve BMP4, Activin A, CHIR99021, IWR-1, and VEGF to induce mesodermal differentiation and cardiac progenitor specification. Vascular cells are derived from hiPSCs using VEGF-A, and SB431542 resulting in CD31 and VE-cadherin expressing endothelial cells. Maturation and functionality of cardiac cell types were determined by several criteria, including contractile force and relaxations measured by analyzing pole deflection, well-defined sarcomere structures visualized by immunostaining for α-actinin and cTnT, and proteomics studies. The latter revealed higher expression levels of mature cardiac markers and endothelial-derived factors like LAMA5 and PDGF-BB, which were critical for extracellular matrix deposition and sarcomeric protein regulation. This approach collectively demonstrates that improved maturation and functionality can be achieved in cardiac organoids when vascular cells are present, compared to non-vascularized organoids (Voges et al., 2023).

In another assembloid approach, Zeltner et al. (2024) recently developed a versatile and modular platform to generate sympathetic neuron-innervated cardiac assembloids (hSCAs), to model sympathetic regulation of the heart. The hSCAs were created by combining hiPSC-derived sympathetic neuron (symN) progenitors with cardiac progenitors. Sympathetic neurons were derived from hiPSCs through a protocol that first differentiated the cells into SOX10+ neural crest cells (NCCs) in a 2D culture with BMP4, SB431542, and CHIR99021, followed by replating the NCCs into 3D spheroids to induce early sympathetic progenitor markers. Cardiac progenitors were derived using a protocol involving CHIR99021 and XAV939 to induce mesodermal differentiation and cardiac specification. These progenitors were then combined in a 1:1 ratio and cultured in floating 3D cultures on a shaker. The maturity and functionality of the hSCAs were evaluated by assessing structural markers like α-actinin and cardiac troponin T (cTnT) through immunofluorescence staining. Functional properties were assessed using video-based analysis and microelectrode arrays (MEAs) for contractility and electrophysiological measurements. The hSCAs exhibited advanced structural and functional characteristics, including the presence of atrial and ventricular specific cells and the formation of chamber-like structures. Polarized patterning for atrial and ventricular markers was observed, with immunofluorescence staining revealing the presence of atrial marker MLC-2a and ventricular marker MLC-2v. Approximately 50% of the hSCAs developed cavity structures resembling heart chambers, with epicardial and endocardial patterning indicated by the exterior layer staining positive for WT1 and the interior layer for NFATC1. Transmission electron microscopy (TEM) revealed mature CM structures of well-aligned myofiber bundles, Z-lines, intercalated discs, and transverse tubules (T-tubules), also confirmed by RT-qPCR analysis revealing high expression levels of MYH7 and MYL2 compared to MYH6 and MYL7. Spontaneous beating and a propagating beating pattern were present. Calcium imaging demonstrated that sympathetic neurons could regulate cardiac activity through neurotransmitter release. Nicotine, a cholinergic receptor agonist, was used to pharmacologically activate the sympathetic neurons, resulting in increased cardiac beating efficiency. Optogenetic stimulation involved using ChR2-expressing neurons activated by blue light, which also enhanced the beating rate. These evaluations highlighted that the addition of sympathetic neurons significantly enhanced the maturation and functionality of cardiac tissues in the hSCAs, resulting in improved contractility, well-defined sarcomeric structures, synchronized electrical activity, and effective regulation of cardiac function through both pharmacological and optogenetic methods.

In summary, the field of synthetic embryology has built from transformative advancements with partially structured cardiac models, such as cardiac organoids and assembloids. These models represent early stages in the progression toward more complete physiologically relevant heart systems, capturing essential cardiac structures and offering insights into basic cardiac biology.

### Synthetic embryology *in vitro* complex heart models

A holistic understanding of heart organogenesis and function beyond a subset of cells and tissues is vital to form a comprehensive view of heart disease, injury and recovery and direct the appropriate therapeutic strategies, including heart organogenesis technology relevant to organ transplantation. Moving to higher levels of complexity poses unique challenges, requiring directed self-assembly, critical cell numbers and the multilineage coordination of diverse cell types to mimic native cardiac development along with extended analysis techniques, including RNA-Seq. One promising approach to achieve this complexity *in vitro* is through gastrulation-based methods (gastruloids) which replicate key anatomic and physiologic events of early heart development without needing full embryogenesis. This capability has been effectively demonstrated in mouse (Rossi et al., 2021) and human (Olmsted and Paluh, 2022) studies, marking a significant step in *in vitro* heart organogenesis (Figure 3C). In the pivotal gastruloid study by Rossi et al. (2021) using mouse embryonic stem cells (mESCs) the authors captured early heart organogenesis events of heart tube formation with *in vivo* like spatiotemporal fidelity of key developmental and structural biomarkers. Stem cell aggregates were exposed to a cocktail of cardiogenic factors, including basic fibroblast growth factor (bFGF), ascorbic acid, and vascular endothelial growth factor 165 (VEGF), to promote cardiac development via Mesp1+ progenitors. These progenitors progressively became restricted to the anterior portion of the gastruloid, forming a cardiac crescent-like domain that elongated and folded into a heart-tube like structure along with beating cardiac tissue. The presence and differentiation of cardiac progenitors was confirmed through advanced methods such as RNAscope imaging, FACS, and single-cell RNA sequencing. RNAscope imaging revealed the spatial localization of biomarkers Mesp1, Nkx2-5, and Tbx5 within the cardiac crescent-like domain. FACS analysis identified increasing populations of Flk1+ and PDGFRα+ cardiac progenitors over time, while single-cell RNA sequencing provided a high-resolution transcriptomic profile, identifying distinct clusters of mesodermal to mature cardiomyocytes and confirming the presence of first and second heart field progenitors. Functional properties were assessed using calcium imaging, which revealed spontaneous, rhythmic calcium spiking in contractile areas, indicative of functional fetal cardiomyocytes. Additionally, a vascular-like network formed, indicated with Flk1+ cells differentiating into CD31^+^ endothelial cells, laying the groundwork for vascular development.

Human organoid and gastruloid technologies benefit greatly from stem cell studies in mouse, including use of neuromesodermal progenitors (NMPs) that define lineages in the trunk including those relevant to organogenesis and multisystem integration (Gouti et al., 2014). Elongating Multi-Lineage Organized Gastruloids (EMLOCs; Olmsted and Paluh, 2022) are the first hiPSC gastruloid model of cardiac organogenesis that reproducibly replicates the cellular and tissue complexity, morphogenic states, chamber genesis, conductive, contractile and electrophysiological maturation, vascularization and extrinsic central and intrinsic cardiac nervous system (CNS-ICNS) integration. The extensively patterned innervation over the multi-chambered, contractile organ mirrors the spatiotemporal organization seen *in vivo* for human and mouse embryonic hearts. Key to multisystem integration in the gastruloid is formation of a polarized hourglass-like structure with balanced domains of cardiogenesis and neurogenesis. EMLOCs were developed by further optimizing elongating multi-lineage organized (EMLO) gastruloids that mimic embryonic gut formation (Olmsted and Paluh, 2021), done via exposure to angiocrine and pro-cardiogenic factors FGF2, VEGF, ascorbic acid (Olmsted et al., 2022; 2023). Gut formation in the human embryo helps establish cues for heart organogenesis and EMLO gastruloids contain biomarkers for heart fields. The heart-centric revised protocol robustly facilitates cardiac organogenesis (Olmsted et al., 2023). Single-cell RNA sequencing at days 7 and 16 post-aggregation revealed the presence of diverse cell types, including cardiomyocytes, epicardial cells, cardiac fibroblasts, neural crest cells, vascular endothelial cells, and neural progenitors and neurons. The data showed distinct cardiac regions expressing markers such as GATA4, GATA6, TBX5, HAND2, TNNT2, and MYL7, indicating the differentiation of first and second heart field progenitors into cardiomyocytes. Immunofluorescence confirmed the presence of cardiac troponin T (cTnT) and sarcomeric structures, along with the formation of a cardiac crescent and heart tube-like structures. Functional assessments through calcium imaging demonstrate rhythmic calcium transients, corresponding to the beating activity of the cardiomyocytes. One of the most striking features of EMLOCs is the progressive innervation of the cardiac regions by neurons, which originate in the neural domain anterior to the primitive gut tube. The spatial patterning observed in EMLOCs mirrors *in vivo* neuronal and cardiac tissues that co-develop in proximity and interact extensively. Neurogenesis was observed to emerge along with SOX2+ and TUJ1+ neural rosettes by day 7, which expand and integrate with the cardiac regions over time. By day 25, elaborate neuronal networks are identified intercalating with the myocardium, resembling ganglionated plexuses that characterize heart innervation. The types of neurons identified included autonomic neurons (ASCL1 and PHOX2B), sensory neurons (POU4F1/BRN3A), and motor neurons (MNX1/HB9). The model also reveals that neurons populate the cardiac regions over time, waiting for chamber genesis to complete, providing a dynamic system to study the spatiotemporal interactions between the CNS-ICNS during cardiac development.

### Unique opportunities for synthetic embryology models in human heart health

Although still in their infancy, human gastruloid technologies for heart synthetic embryology are expected to have profound implications for human heart health, providing more holistic organ-based responses to injury and disease. Immediate therapeutic benefits from synthetic embryology models may come in the use of these heart models to provide alternate optimal sources for cardiac cells, including CMs and pacemaker cells, or tissues such as valves, and to facilitate goals to achieve full size organs. The knowledge on how to integrate neuronal elements into a heart synthetic embryology model provides new opportunities surrounding arrhythmogenic diseases and spinal cord injury impacts. Congenital heart disease will benefit from foundational organoid and assembloid models as well as gastruloid synthetic embryology models. Here, we briefly overview these three areas.

### Neuronal function in arrhythmogenic diseases

A variety of mechanisms have potential to disrupt the normal electrical conduction and contraction patterns of the heart and lead to severe arrhythmias, impacting overall heart function and increasing the risk of sudden cardiac death. Synthetic embryology heart models with neuronal integration offer a unique platform to study the pathophysiology of these diseases in a controlled environment, allowing researchers to observe how arrhythmogenic conditions develop and progress at the cellular and tissue levels (Maurissen et al., 2024). Neural input plays a critical role in the regulation of heart rhythm and function. Abnormalities in neural input can lead to various cardiac diseases, each with distinct mechanisms and consequences. For example, the autonomic nervous system plays a crucial role in the genesis and maintenance of atrial fibrillation (AF), the most common arrhythmia, with simultaneous sympathetic and parasympathetic nerve activity often preceding its onset. Studies have shown that while both extrinsic and intrinsic cardiac autonomic nervous systems contribute to AF, intrinsic cardiac nerve activity alone can sometimes trigger AF (Patterson et al., 2006; 2007; Choi et al., 2010). Cardiac gastruloid models and cardiac organoids and assembloids are providing new resources for detailed studies of disease mechanisms underlying arrhythmogenic diseases as well as genetic drivers, sex and ethnic contributions from congenital to adult. These models can be manipulated to mimic various genetic and environmental factors that contribute to arrhythmias including ion channel mutations, such as those in potassium channels (KCNQ1 and KCNH2) and sodium channels (SCN5A), associated with Long QT Syndrome (LQTS) and Brugada Syndrome and oxidative stress, from excessive reactive oxygen species (ROS) production, which damages cellular components, including ion channels and mitochondria, leading to altered calcium handling and electrical instability contributing to the development of AF and heart failure-related arrhythmias by promoting atrial remodeling, fibrosis, and exacerbating electrical instability (Shen and Zipes, 2014).

### Spinal cord injury impacts on the central and intrinsic cardiac nervous system (CNS-ICNS)

The ICNS plays a crucial role in regulating heart rate and contractility, and its disruption due to spinal cord injury (SCI) can result in hypotension, orthostatic hypotension, and cardiac dysrhythmias. High-level SCI, specifically those injuries at or above the thoracic spinal segment 6 (T6), cause reduced cardiac inotropic function and altered cardiac loading leading to significant reductions of left ventricular chamber size and mass, which in turn lead to altered indices of systolic and diastolic function (Williams et al., 2018; Fossey et al., 2022). These spinal injuries also often result in hypotension or low resting blood pressure, which can persist for years following the injury (Mathias et al., 1979; Krassioukov and Claydon, 2006). Cardiac dysrhythmias, including bradycardia or slowed heartbeat, are prevalent, particularly in the acute phase of SCI, within the first week post-injury (Mathias, 1976; Winslow et al., 1986; Lehmann et al., 1987). The irregular heartbeats initiated by high-level spinal injuries are initially more serious but tend to diminish as the injury progresses to chronic (Krassioukov et al., 2007). EMLOCs utilize neuromesodermal progenitors that are the natural precursor to human trunk lineages. This benefits modeling the impacts of SCI on the ICNS and heart function in this and future synthetic embryology heart gastruloid models, and is a potentially powerful approach to study these complex interactions, perturbations and drug treatments.

### Complex heart models to inform on congenital heart disease

Congenital Heart Disease (CHD) encompasses structural abnormalities of the heart present at birth with differing functional health impacts and are the most common human birth defect. Mutations in several key genes that regulate critical steps in heart formation, such as TBX5, NKX2-5, and GATA4, can lead to chamber septation anomalies and impact the heart conduction system. Dysregulation of signaling pathways for Notch and Wnt also impacts valve formation and outflow tract development, including altered hemodynamic forces during early heart morphogenesis that compound structural defects (Bruneau, 2008). Severe CHD includes life-threatening defects, such as oxygen deprived cyanotic conditions like tetralogy of Fallot and hypoplastic left heart syndrome. Also severe are acyanotic conditions, such as large ventricular septal defects and severe aortic stenosis, which impair blood flow. Moderate CHD generally centers around defects of valve stenosis or mild aortic narrowing. Mild CHDs are most common and are asymptomatic or self-resolving, including small ventricular septal defects, patent ductus arteriosus, and bicuspid aortic valves (Hoffman and Kaplan, 2002). Human cardiac organoids have already shown success in modeling physiology and electrophysiology changes in response to metabolic impacts of pregestational diabetes (Lewis-Israeli et al., 2021), as well as impact of the drug ondansetron on CHD (Volmert et al., 2023). Expanded scaled, multi-omic, and bioengineered studies of CHD in human organoid and gastruloid models will benefit from studies in mouse (Rao et al., 2022) and continue to in parallel drive new technologies for 3D *in vitro* analysis. This ability to investigate genetic, environmental, and biomechanical influences on critical stages in heart development opens the door for new insights into CHD causes, interventions and treatments.

## Discussion

Human cardiac stem cell research has seen transformative progress, with stem cell-derived cells, tissues, organoids, assembloids and gastruloid synthetic embryology models offering progressively refined perspectives on heart function, tissue organization, multistage development, and disease. Extending from hiPSC-CM studies a succession of increasingly complex foundational models, each contributing unique insights into distinct mechanisms of heart development and advancing our ability to recreate the heart in its intricate structure and function, have been developed. Self-organizing human cardiac microchambers were among the first models to introduce confined beating microchambers, offering a simplified system to study early cardiac tissue organization and contraction. Next followed 3D cardiac microtissues (MTs), which incorporated multiple cardiac cell types and began to capture the structural and functional properties of mature cardiomyocytes, establishing a foundation for exploring cellular interactions in a multi-dimensional context. Heart-forming organoids (HFOs) expanded on 3D tissues include endocardial-like cells, septum-transversum-like structures, and a vascular network, bringing the first glimpse into multi-lineage integration and spatial organization. With the development of cardioids, self-organizing chamber-like structures became possible, allowing studies of internal cavity formation and myocardial layer patterning. Self-assembling human heart organoids (hHOs) moved the field further by including endocardial-lined chambers and a network of vascular endothelial cells, enabling research into atrioventricular specification and heart field formation. Human-chambered cardiac organoids (CCOs) added internal chambers with organized sarcomeres, providing more intricate models for structural heart development. The introduction of patterned self-organizing human primitive heart organoids brought both atrial and ventricular chambers into a single model, adding anterior-posterior patterning and proepicardial organ formation, which advanced our understanding of chamber specificity. Human vascular cardiac organoids (hVCOs) introduced vascular and fibroblast elements, enhancing cardiomyocyte maturation and underscoring the importance of vascular integration. Sympathetic neuron-innervated heart assembloids (hSCAs) incorporated sympathetic neurons, allowing researchers to study the regulation of cardiac rhythm by neural inputs, crucial for understanding arrhythmogenic conditions. These partially structured heart models focus on specific elements of heart function but are unable to address the full interactive complexity of a full heart model.

Heart gastruloid technologies represent a significant advancement in synthetic embryology, integrating endoderm, mesoderm, and ectoderm lineages into a holistic model of heart organogenesis as a subset of the embryonic process *in vitro*. The heart, as the first organ to develop, emerges alongside the CNS, guided by a spatiotemporal interplay of genes and signaling pathways that establish the foundational cues for organ positioning and morphogenesis. Two prominent gastruloid models capture this complexity: the mouse model by (Rossi et al., 2021)and the human model by (Olmsted and Paluh, 2022). Both models leverage a polarized structure that enables the co-development of pathways critical for organogenesis and tissue integration. The mouse gastruloid model by (Rossi et al., 2021) reproduces early cardiogenesis forming a cardiac crescent and elongating into a heart tube-like structure with beating regions and vascular networks. The human Elongating Multi-Lineage Organized Cardiac (EMLOC) gastruloid is the most advanced synthetic embryology cardiac model to date, with steps from early cardiogenesis, through chamber genesis and vascular and neuronal integration. Guidance cues for the integration of vascular and neural components with chamber-like structures are present in EMLOCs as revealed by patterning that mimics that of the natural mouse and human hearts.

Detailed RNA-Seq profiles of the mouse gastruloid model by (Rossi et al., 2021) and the human EMLOC model by (Olmsted and Paluh, 2022) highlight their cellular complexity and ability to replicate early cardiogenesis and for EMLOCs later developmental stages and neurogenesis. In the mouse model, distinct cardiac progenitors, including first and second heart field cells (Mesp1+, Nkx2-5+, Tbx5+), and endothelial cells (Flk1+, CD31^+^) were identified, supporting the formation of cardiac crescent-like structures and vascular networks. The human model revealed an even broader cellular repertoire, including cardiomyocytes (TNNT2+, MYL7+), neural crest cells (SOX10+), and neurons, such as autonomic (ASCL1+, PHOX2B+), sensory (BRN3A+), and motor neurons (HB9+). Functional studies in both models demonstrated rhythmic calcium transients indicative of cardiomyocyte contractility, while immunofluorescence confirms sarcomeric organization. Both gastruloid heart models represent significant milestones as high complexity platforms. EMLOCs offer the unique opportunity to study the cues necessary for spatiotemporal coordination of cardiogenesis with neurogenesis along with congenital heart disease mechanisms that may require a more complete heart model. The ability to integrate tri-lineage cellular diversity, morphogenic events and developmental pathways appropriate for embryological cardiogenesis positions synthetic embryology gastruloid models as groundbreaking tools in understanding human heart development, injury and disease.

## Future outlook

The potential for synthetic embryology to contribute to regenerative medicine is extensive. While developing full-sized organs for transplantation *in vitro* through self-assembly remains a longer-term goal, intermediate steps such as generating functional cardiac tissues, valves, and pacemaker cells for therapeutic purposes are within reach. These advancements could revolutionize the treatment of heart diseases, offering new hope for patients requiring cardiac repair or replacement. Development of precise models of patient-specific heart conditions, including congenital defects, will be enabling for customized treatment plans and personalized medicine approaches. Additionally, advanced synthetic embryology heart models with multisystem input, such as vascularization and neurogenesis may enable multiorgan system models to emerge. A challenge to synthetic embryology heart models is the need for codevelopment of advanced bioengineering platforms, including multimodal sensing, to drive data collection and innovation. This will further enable integration of these models with advanced computational tools and machine learning predictive models of heart disease progression and treatment outcomes. Ultimately, the continued advancement of cardiac synthetic embryology models holds the promise of transforming our approach to understanding, diagnosing, and treating not only heart disease and cardiovascular medicine, but paving the way for a new era of organ-based models in medicine.
